# Organ‐Specific Proteomic Aging Clocks Reveal Complexities Underlying the Healthy Drinking Paradox

**DOI:** 10.1111/acel.70743

**Published:** 2026-09-24

**Authors:** Gabin Drouard

**Affiliations:** ^1^ Institute for Molecular Medicine Finland (FIMM), HiLIFE Helsinki Finland

## Abstract

The “healthy drinking” paradox refers to situations where alcohol consumption is misleadingly associated with better health outcomes, a pattern frequently observed in observational studies that are prone to a range of confounding factors. Some recent studies developing organ‐specific proteomic aging clocks report that alcohol consumption is linked to slower aging in select organs, even as it accelerates aging in others, thus echoing this paradox, with substantial discrepancies across such studies. In this Perspective, I summarize findings to date, discuss potential reasons for these contradictory results, such as differences in clock construction, alcohol phenotyping, and confounding control across studies, and highlight both the limitations and promise of organ‐specific clocks for studying epidemiologically challenging traits such as alcohol consumption. Taken together, these findings call for careful interpretation of observational studies linking alcohol consumption to systemic and organ‐specific biological aging, which may, if taken at face value, understate the known damage that alcohol causes to human health. Further work comparing organ‐specific clocks developed across different studies from a biological standpoint is needed to fully decipher the true effects of alcohol consumption, alongside research applying aging clocks to alcohol consumption within more advanced epidemiological designs and across different dimensions of aging (chronological age vs. mortality).

## Graphical abstract

Organ‐specific proteomic aging clocks suggest alcohol consumption is linked to slower aging in some organs while accelerating it in others, echoing the “healthy drinking” paradox. This Perspective examines the reasons for such contradictory results across studies, and argues for cautious interpretation alongside further cross‐study and methodological work.

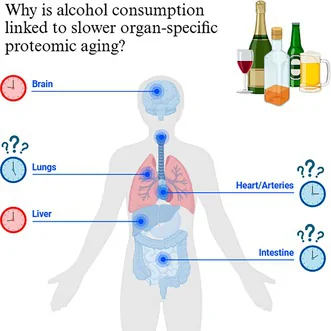

Heavy alcohol consumption is harmful to health, notably contributing to earlier cardiovascular disease onset and organ damage, particularly of the liver (Piano et al. 2025). While the deleterious effects of alcohol on population health are well documented, observational studies have sometimes reported paradoxical findings, highlighting the complexity of alcohol consumption as an epidemiological exposure. This “healthy drinking” paradox refers to the seemingly protective effect of alcohol use on health. For instance, studies assessing the relationship between alcohol consumption and body weight indicate that although alcohol is caloric, alcohol consumption is sometimes reported to be negatively associated with body weight, notably in women (Siegmann et al. 2022). This may be partly due to factors such as reduced food intake or co‐occurring conditions (e.g., smoking or mental health issues) among heavy drinkers. Another example concerns mortality risk, as low‐volume alcohol drinkers do not exhibit higher mortality than abstainers (Zhao et al. 2023). This likely reflects the heterogeneity of abstainers, among whom alcohol avoidance may be motivated by past family issues, previous alcohol dependence, or medical advice, suggesting that some individuals refrain from drinking because their health is already compromised. On the other hand, very low levels of drinking may not affect risk of dying sufficiently to cause an observable difference in risk, given long average time between exposure and death. Overall, this paradox highlights the challenges of addressing confounding in observational studies of alcohol consumption, which complicates efforts to quantify the true harm of alcohol and to understand the mechanistic processes through which alcohol contributes to disease. Thus, studies on the pathophysiology of alcohol have focused on immediate physiological and metabolic effects, which can be considered biomarkers of use and abuse. Further, existing biomarkers such as DNA methylation or metabolites do not assess multiple organs and metabolic systems comprehensively.

Recently, aging clocks built on proteomic data have shown promise for studying complex traits and have offered new avenues to investigate aging at the organ level, which may help understand alcohol‐related harm to the body and its organs. Proteomic aging clocks are models that use proteins as predictors to estimate an individual's biological age. While chronological age measures the time elapsed since birth, biological age reflects physiological state, with a greater biological age relative to chronological age referred to as accelerated aging. It is worth noting that aging clocks may be trained to predict chronological age or mortality risk, thus capturing different aspects of aging; hereafter, I refer to aging clocks regardless of the training outcome. The recent development of organ‐specific proteomic aging clocks adds the ability to evaluate aging in individual organs, based on the principle that aging does not occur uniformly across tissues (Oh et al. 2023), in contrast to systemic clocks that estimate biological age at the whole‐organism level. The value of organ‐specific clocks lies in their potential to predict organ‐specific diseases. For example, Goeminne and colleagues demonstrated that their newly developed kidney‐specific clock excelled at predicting kidney failure, while a lung‐specific clock best predicted chronic obstructive pulmonary disease (Goeminne et al. 2025).

Regarding alcohol consumption, recent findings using organ‐specific clocks have produced puzzling results and further illuminate the “healthy drinking” paradox. Figure 1 presents key findings from these studies, along with details of their designs. In the study by Goeminne and colleagues, alcohol consumption was positively associated with increased mortality risk according to the brain, kidney and intestine clocks, highlighting the harmful effects of alcohol consumption (Goeminne et al. 2025). However, the same study also reported negative associations with lung‐ and artery‐specific clocks. In another study developing different organ‐specific aging clocks, the authors observed negative associations between alcohol consumption and the kidney, lung, artery, muscle and intestine clocks (Wang et al. 2026). While this study supports Goeminne et al. by demonstrating decreased biological aging at the lung and artery levels in individuals with higher alcohol consumption, consistent with the decreased mortality risk reported by Goeminne et al., it also contrasts with their findings by suggesting that associations between alcohol consumption and kidney or intestine aging may be positive rather than negative. A third study provided further insights into how alcohol consumption is reflected in aging across different organs (Wang et al. 2025). In contrast to the two other studies, the authors observed that alcohol consumption was associated with faster aging across most of the organs they investigated, except the intestine clock, which was negatively associated with alcohol consumption but did not pass multiple testing corrections. Interestingly, the authors found that alcohol consumption with meals had a “protective” effect on aging across multiple organs, with metabolic timing, modulation of the gut microbiota, or organ‐specific regulation of inflammatory responses suggested as plausible pathways explaining these results (Wang et al. 2025). This finding may, however, also reflect different aspects of drinking, whereby consuming alcohol during meals is more likely to be a social and moderate activity, which may explain the less deleterious effects reported, although the reversal of association directions may raise questions regarding potential confounding effects. Confounding effects from socio‐economic background are indeed likely, as supported by additional findings of Goeminne et al. who reported that wine consumption, often considered a higher class activity, appeared to be “protective” of organ aging, whereas beer drinking, likely reflecting alcohol consumption in lower socio‐economic groups, was associated with accelerated aging independent of covariates including the Townsend deprivation index (Goeminne et al. 2025).

**FIGURE 1:**
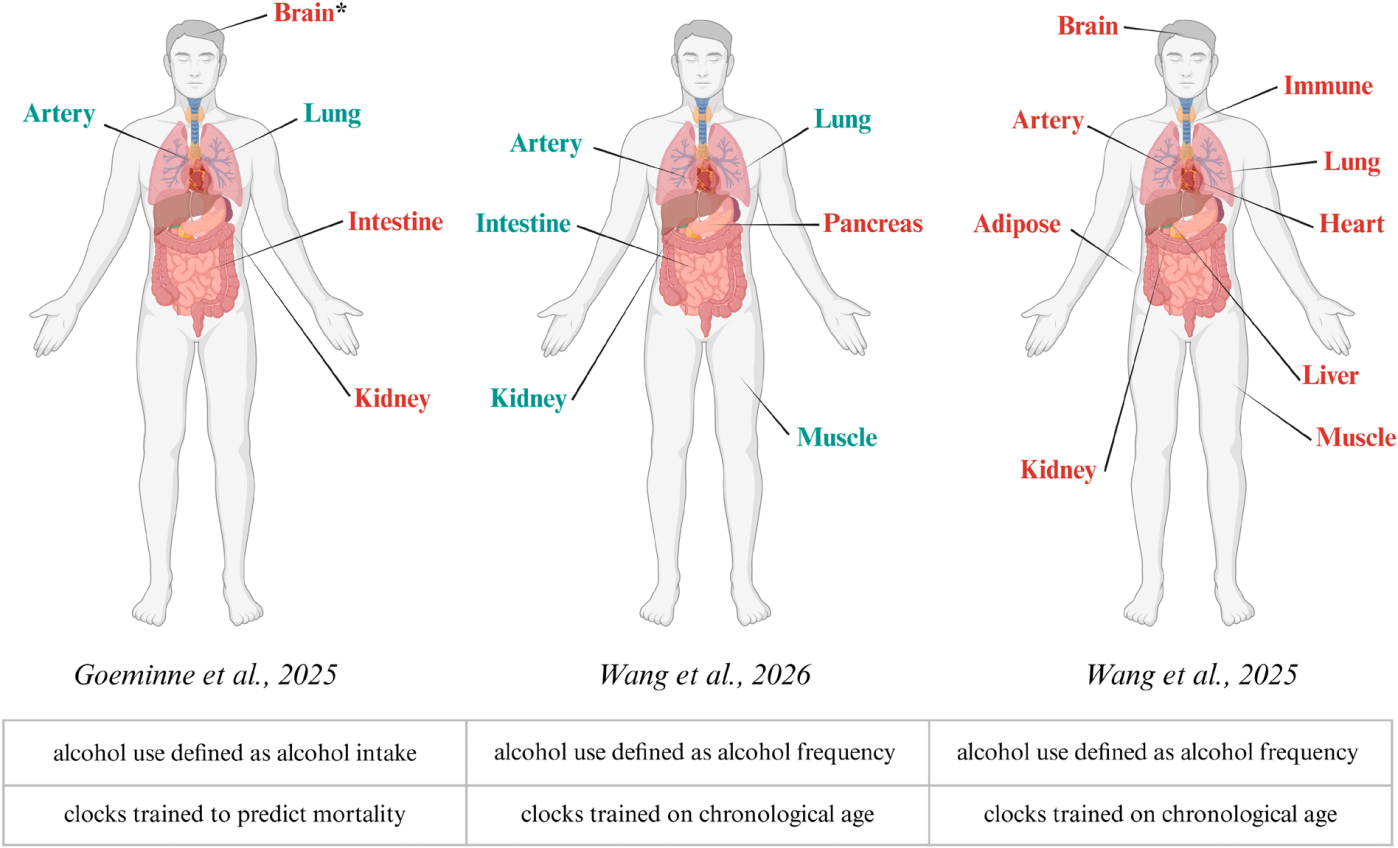
Reported associations between alcohol consumption and organ‐specific clocks across three studies. Organs in red indicate those for which the organ‐specific clocks were positively associated with alcohol consumption after multiple testing correction (*p* < 0.05), while organs in green indicate negative associations. All three studies are based on UK Biobank Pharma Proteomics Project (UKB‐PPP) data (Sun et al. 2023), where the targeted population consists of approximately 50,000 adults aged from the late 30s to 70s; however, the studies differ in methods. In Goeminne et al. (2025): Clocks are trained to predict mortality; alcohol consumption is defined as alcohol intake (grams/day); multiple testing correction is FDR based; and model adjustment includes sex, age, Townsend deprivation index, physical activity, and smoking status. In Wang et al. (2026): Clocks are trained on chronological age; alcohol consumption is defined as alcohol frequency (daily or almost daily vs. < 4 times per week); multiple testing correction is FDR based; and model adjustment includes sex, age, Townsend deprivation index, physical activity, and smoking status. In Wang et al. (2025): Clocks are trained on chronological age; alcohol consumption is defined as alcohol frequency; associations were adjusted for sex and age only, with a more stringent Bonferroni correction. The * symbol indicates an association for which the corrected *p*‐value, while not below 0.05, was below 0.10.

Altogether, these studies illustrate the “healthy drinking” paradox, suggesting slower aging in some organs among individuals who consume more alcohol, yet with notable inconsistencies across studies. These inconsistencies may arise from differences in the protein sets used to construct the clocks despite all being based on the Olink platform. There are also differences in alcohol consumption definitions (e.g., total intake versus drinking frequency), and in the outcomes, whether biological age or mortality, on which the clocks were trained (Figure 1). However, two of the three studies discussed here, namely Wang et al. (2026, 2025), both trained their organ‐specific clocks on chronological age in the UK Biobank, and the aging clocks‐alcohol associations reported in Figure 1 all arise from alcohol consumption being defined as alcohol frequency. This indicates that the large discrepancies across these two studies likely originate from the protein set utilized, and yet stronger adjustment for socioeconomic traits in Wang et al. (2026) cannot be ruled out as a reason for these discrepancies. As reflected in Wang et al. (2025), where alcohol consumption was associated with aging clocks in opposite directions depending on whether alcohol was consumed during meals, adjustment for socioeconomic factors is warranted and commendable.

Beyond methodological explanations, biologically divergent mechanisms may contribute to the observed discrepancies across studies. Echoing known differences between systemic clocks in the literature, different organ‐specific clocks likely capture distinct biological processes rather than a single underlying aging construct, depending on the outcome on which each clock was trained. One example is that of gut microbiota modulation. Polyphenol‐rich beverages such as wine can shift microbial composition and reduce markers of systemic inflammation (Buljeta et al. 2023), which may partly explain the association observed in Goeminne et al. (2025) between wine consumption and slower organ aging, in contrast to beer consumption, which was linked to faster aging. However, confounding lifestyle and dietary factors are likely the main drivers of the observed differences between wine and beer consumption. In the particular case of brain aging, the brain clock's synaptic signature is consistent across both Goeminne et al. (2025) and Wang et al. (2026), with both reporting enrichment in synapse‐related pathways (for Goeminne et al. (2025), per pathway analyses in the supplement of David Sarmento et al. (2026)); yet alcohol consumption was associated with brain aging in Goeminne et al. (2025) but not in Wang et al. (2026). This suggests that, in this particular case, the inconsistency likely arises from methodological differences (e.g., alcohol intake vs. alcohol frequency as the exposure definition) rather than from biological divergence. Studies directly comparing the functional underpinnings of organ‐specific clocks are needed to disentangle methodological from biological sources of inconsistency. Finally, because organ‐specific proteomic clocks rely on protein weights estimated independently of alcohol exposure, associations between alcohol consumption and clock‐based aging estimates may partly reflect the alcohol sensitivity of individual proteins rather than a true effect on organ aging, which may also contribute to discrepancies across studies.

Despite known limitations of organ‐specific aging clocks in their generalizability, biological interpretability, and translational utility (Xiao et al. 2026), they may still provide opportunities to investigate the biological mechanisms through which alcohol contributes to disease (Wyss‐Coray and Topol 2026). These opportunities remain, however, largely unexplored, and the current literature, recent as it is, presents findings whose interpretations and potential implications are yet to be fully understood. Further research is therefore needed, including implementation across diverse settings and populations. Investigating alcohol consumption and organ aging through a longitudinal design may allow greater control for confounding effects and facilitate the establishment of near‐causal scenarios. That said, longitudinal designs may still be prone to biases such as the sick‐quitter bias, where individuals' alcohol consumption may stop or decrease drastically because of medical recommendations. Examining clock‐alcohol associations longitudinally may thus be optimal in environments where medication use, purchase, and prescription information is available together with proteomic data, as in biobanks such as FinnGen (Kurki et al. 2023), so as to limit the influence of alcohol changes initiated by recent medical recommendations. Using different organ‐specific clocks simultaneously may also enable a better understanding of why organs may show accelerated or decelerated aging depending on the clock used, potentially in combination with newly developed organ‐specific epigenetic clocks (Sehgal et al. 2025), thereby providing an additional biological layer. As such, the epigenome, whose marks are partially reversible, may better capture long‐term imprints of alcohol consumption than the proteome, the latter being a snapshot of protein activity at a given timepoint; studies comparing epigenetic to proteomic clocks are, however, needed. The role of alcohol consumption across different dimensions of aging also remains to be elucidated, as its association with mortality at a molecular level may differ substantially from its effects on the aging process. Finally, refining our understanding of the determinants driving these associations, that is, the extent to which genes or environmental exposures influence them, may also provide important insights. In this respect, twin and family studies may be particularly informative, given their methodological ability to examine genetic and environmental effects and the extensive information on substance use acquired through the historical focus on behavioral traits within this research community (Agrawal et al. 2012).

## Author Contributions

G.D. wrote the manuscript.

## Funding

The author has nothing to report.

## Conflicts of Interest

The author declares no conflicts of interest.

## Data Availability

Data sharing not applicable to this article as no datasets were generated or analysed during the current study.
